# Rathkeale Infirmary

**Published:** 1833-07

**Authors:** 


### RATHKEALE INFIRMARY.

*Cases of Hemeralopia.* By Dr. Patterson. [*Dublin Journal of Med. and Chem. Sciences.*]

Case I. James Dwyer, aged nineteen years, applied at the Rathkeale Infirmary, the 9th September, 1831. He complained that every evening at dusk he became almost blind, and continued so till sunrise next morning; that he could see very imperfectly by fire or candle light, and that exposure to heat and active exercise, or labour, always considerably aggravated his inability to discern objects at night. He stated, that in candlelight, however vivid, he could distinguish no object that was directly opposite to him. He could see a little in an oblique direction, and this enabled him, with much difficulty, to grope his way. The pupils were permanently and very much dilated. "He felt stupid and confused in his head." There was great fulness, hardness, and some tenderness of the right hypochondrium; conjunctiva yellowish; alvine evacuations white. Early in May, 1831, he was attacked with jaundice. He did not get rid of it for a month, and, while labouring under the disease, he first noticed the derangement of sight. This continued, with only daily intermissions, up to the period of his application.

Having been bled, blistered, and purged, with little effect, he was directed to rub in one drachm of strong mercurial ointment every night, and, as soon as this had affected the constitution, recourse was had to the nitro-muriatic acid bath. The moment the gums became tender, he experienced a manifest improvement of his sight. But at this time he complained, for a few days, that exposure to artificial light produced most unpleasant sensations in his eyes. Under the treatment, the hepatic disorder was quickly and effectually relieved, and with it the amaurotic affection entirely disappeared.

Case II. In November, 1831, Patrick Mangan, aged thirty-two, applied for relief, and stated that, "about six weeks before, he was affected with jaundice, which continued for eight or ten days, but having been badly cured, it fell on his eyes. Ever since he is unable to see any object at night, however bright it may be; neither can he see in a lighted room." There was pain and fulness of the right hypochondrium; dilated pupils; yellow tinge of the conjunctiva; and high coloured urine with pink sediment.

He rubbed in Ung. Hyd. fort. ʒi. every night, used the nitro-muriatic acid bath, and took an occasional dose of calomel and cathartic extract at night, followed by infusion of senna and sulph. magnesiæ in the morning. Through these means his amendment was rapid. In ten days he was able to read the smallest print by candlelight, and became entirely free from hemeralopia.

Case III. The 14th December, 1831, James Wynn, aged eighteen years, became a patient at the Rathkeale Infirmary. He complained of languor and loss of appetite. Skin and conjunctiva yellow; alvine evacuations white; urine high-coloured, resembling beer; no pain of side or epigastrium on pressure. He first noticed the yellowness of skin three weeks before his application, and from that period, he stated, he was regularly attacked with almost perfect blindness at dusk every evening. It continued the whole night, and disabled him from going about, unless led by some person. His sight was natural in the daytime. Objects did not even appear of a yellowish hue, as they have been said to do to jaundiced patients. The pupils were very much dilated; bowels costive.

His bowels were briskly moved every morning for five or six days, by pulv. jal. comp. and calomel, with the effect of diminishing the jaundiced state of the skin, but without the least benefit to his nocturnal loss of vision. Recourse was then had to the constitutional effects of mercury; and the system having been brought under the influence of that mineral, he completely recovered his usual acuteness of sight, in discerning objects at night.

In the present year, I have also met with other cases of hemeralopia, but they were much slighter in degree than those which have been related. The subjects were generally males. Two were at the time labouring under slight jaundice of a few days' continuance. The others had lately recovered from a similar affection. In all these cases the nitro-muriatic acid bath was employed; and, as it was found effective in producing permanent cures, no attempt was made to induce mercurial constitutional action.

Since my attention has been directed to the connexion which appears to exist between hemeralopia and jaundice, I have observed, in every case of the latter complaint that has fallen under my observation, more or less tendency to the former affection. In all there existed preternatural and fixed dilatation of the pupils; and when questions were put concerning the state of vision, in most instances, complaints occurred, in some degree, of its nocturnal impairment.

